# Point of Care Liquid Biopsy for Cancer Treatment—Early Experience from a Community Center

**DOI:** 10.3390/cancers16142505

**Published:** 2024-07-10

**Authors:** Champica Nicholas, Andrea Beharry, Anna M. Bendzsak, Kassandra R. Bisson, Keith Dadson, Shaan Dudani, Marco Iafolla, Kashif Irshad, Kirstin Perdrizet, William Raskin, Raviya Singh, David Chun Cheong Tsui, Xin Wang, Ching Yeung, Parneet K. Cheema, Brandon S. Sheffield

**Affiliations:** 1 Osler Research Institute for Health Innovation, William Osler Health System, Brampton, ON L6R 3J7, Canadaparneet.cheema@williamoslerhs.ca (P.K.C.); 2 Division of Advanced Diagnostics, William Osler Health System, Brampton, ON L6R 3J7, Canada; 3 Division of Thoracic Surgery, William Osler Health System, Brampton, ON L6R 3J7, Canada; 4 Thermo Fisher Scientific, Burlington, ON L7L 5Z1, Canada; 5 Division of Medical Oncology, William Osler Health System, Brampton, ON L6R 3J7, Canada; 6 Department of Medicine, University of Toronto, Toronto, ON M5S 1A1, Canada; 7 Division of Medical Oncology, Scarborough Health Network, Scarborough, ON M1P 2V5, Canada; 8 Division of Medical Oncology, UHN Princess Margaret Cancer Centre, Toronto, ON M5S 1A1, Canada

**Keywords:** cancer, biomarker, liquid biopsy, next-generation sequencing (NGS), lung, colon, breast, pathology, oncology, precision medicine

## Abstract

**Simple Summary:**

Cancer care is being increasingly driven by biomarker testing. Many biomarkers are traditionally measured on a sample from a patient tumor such as a biopsy. More recently, “liquid biopsy” has emerged as a blood test to complement or replace traditional tissue-based testing. In this report, we explore one of the first case series of rapid liquid biopsy performed within a hospital setting. The results include a median 3-day time to results, compared to approximately 14 days using traditional centralized reference labs. Additional details of the cohort are shared to highlight the utility of point of care liquid biopsy.

**Abstract:**

Liquid biopsy is rapidly becoming an indispensable tool in the oncologist’s arsenal; however, this technique remains elusive in a publicly funded healthcare system, and real-world evidence is needed to demonstrate utility and feasibility. Here, we describe the first experience of an in-house point of care liquid biopsy program at a Canadian community hospital. A retrospective review of consecutive cases that underwent plasma-based next-generation sequencing (NGS) was conducted. Liquid biopsy was initiated at the discretion of clinicians. Sequencing followed a point of care workflow using the Genexus™ integrated sequencer and the Oncomine precision assay, performed by histotechnologists. Results were reported by the attending pathologist. Eligible charts were reviewed for outcomes of interest, including the intent of the liquid biopsy, results of the liquid biopsy, and turnaround time from blood draw to results available. A total of 124 cases, with confirmed or suspected cancer, underwent liquid biopsy between January 2021 and November 2023. The median turnaround time for liquid biopsy results was 3 business days (range 1–12 days). The sensitivity of liquid biopsies was 71%, compared to tissue testing in cases with matched tissue results available for comparison. Common mutations included *EGFR* (29%), in 86 lung cancer patients, and *PIK3CA* (22%), identified in 13 breast cancer patients. Healthcare providers ordered liquid biopsies to inform diagnostic investigations and treatment decisions, and to determine progression or resistance mechanisms, as these reasons often overlapped. This study demonstrates that rapid in-house liquid biopsy using point of care methodology is feasible. The technique facilitates precision treatment and offers many additional advantages for cancer care.

## 1. Introduction

Biomarker-directed therapy represents the current standard of care in the treatment of many types of cancer [1,2]. Providing personalized therapies can improve treatment efficacy and limit side effects often associated with conventional therapies, improving the quality of life and care of cancer patients [2,3,4].

Comprehensive molecular profiling, using next-generation sequencing (NGS), has proven to be valuable in the provision, and development, of targeted therapies. Using multi-gene panels, NGS can identify genomic aberrations and oncogenic drivers in a variety of cancers [1,5,6,7]. In several disease sites, optimal treatments are determined by the sum of multiple different biomarker results [8,9,10,11].

Tissue-based testing is considered the gold standard in biomarker testing practices; however, there are barriers to obtaining, accessing, and utilizing such samples [1,3,6]. Tissue biopsies are invasive, risky, and time-consuming, which can potentially delay treatment. Moreover, the sample itself may not reflect the biology of the tumor due to spatial or temporal heterogeneity [1,2,3].

Plasma-based molecular profiling of circulating tumor molecules (liquid biopsy) presents an opportunity to interrogate tumor-specific biomarkers and inform clinical care without the associated encumbrances of tissue biopsy [7,12]. Liquid biopsy refers to the analysis of circulating biomarkers shed by tumor cells, such as cell-free nucleic acids, circulating tumor cells, proteins, and nucleosomes. These biomarkers are found in bodily fluids including blood, urine, or saliva [1,2,3,7,12]. This paper refers to liquid biopsy as the profiling of circulating tumor DNA/RNA (ctDNA, ctRNA) collected from a peripheral blood draw—a minimally invasive, safe, repeatable, and time-efficient procedure [2,7,13]. These circulating DNA/RNA fragments provide similar information to nucleic acids extracted from a tissue biopsy; with additional relevant information including a quantitative measure of systemic tumor burden [3,12]. Compared to traditional biopsies, liquid biopsy may alleviate the necessity for procurement of tumor tissue, expand access to precision care, and increase accuracy, while decreasing risk to patients and costs to the healthcare system [12,14,15].

Many health systems are faced with challenges in delivering timely and comprehensive biomarker testing. Notable barriers include tissue availability, turnaround time, and cost, among others [2,3]. While liquid biopsy may offer much assistance in overcoming these barriers, the technique is not widely utilized in publicly funded healthcare systems [16]. At the time of this report, liquid biopsy is available through many commercial vendors. The technique is not currently reimbursed in most publicly funded systems, and most hospitals in these regions do not provide in-house liquid biopsy testing. Testing may be accessed only by private-pay, insurance coverage, or other means such as through clinical trials [16]. Despite the promising evidence of liquid biopsy to provide biomarker information at less cost to the healthcare system, its adoption in clinical practice is limited [2,3,7].

Our group has previously implemented rapid in-house or *point of care biomarker testing*, with an ensuing median turnaround time of 3 business days [17,18]. In parallel to establishing point of care tissue-based testing, our group implemented a rapid in-house point of care liquid biopsy service. In this report, we describe the early clinical experience using point of care liquid biopsy to treat cancer patients in a community setting.

## 2. Materials and Methods

### 2.1. Liquid Biopsy Implementation

Liquid biopsy using the Oncomine precision assay and Genexus™ integrated sequencer (Thermofisher, Waltham, MA, USA) was established in January 2021. The assay was validated using a combination of commercial controls, as well as archived plasma samples with matched results from various commercial liquid biopsy assays, showing a high degree of concordance. Following validation, the assay was made available to clinicians at William Osler Health System, Canada, without restriction. Educational sessions were held for medical oncology staff, as well as nursing teams in the oncology inpatient wards and ambulatory clinics. Additional information was provided on an as-needed basis to other specialists including surgeons, respirologists, hospitalists, emergency room physicians, and nurse practitioners. Liquid biopsies were initiated by clinicians for any indication of their choosing. Plasma isolation and sequencing were performed by medical laboratory assistants and immunohistochemistry technologists. All NGS results were interpreted and reported by a molecular pathologist.

### 2.2. Next-Generation Sequencing of ctDNA/ctRNA

First, 10 mL of peripheral blood was collected in two 5 mL Streck cell-free DNA blood collection tubes. All samples were drawn in-house and received within the lab directly after phlebotomy. No courier services or ambulatory collection was utilized during this period. Upon receipt in the laboratory, the peripheral blood samples were centrifuged at 4000 rpm for 10 min at 4 °C. The plasma portion was aliquoted from the spun sample into DNase/RNase-free Eppendorf microtubes and centrifuged once more at 16,000 rpm for 10 min at 4 °C. The plasma was immediately subjected to nucleic acid extraction, and if not immediately extracted, the supernatant plasma was aliquoted into 2 mL cryogenic tubes and frozen at −80 °C.

Combined cell-free DNA and RNA (cfTNA) extraction was performed with the MagMAX cell-free total nucleic acid isolation kit (Thermofisher, Waltham, MA, USA). Following the manufacturer’s protocol, extraction was performed on plasma from 2 mL or 4 mL volumes depending on the plasma yield. Plasma digestion was performed with proteinase K incubated at 65 °C for 30 min. The digested plasma was then transferred to a processing plate for nucleic acid isolation using the KingFisher Duo Prime Magnetic Particle Processor (Thermofisher, Waltham, MA, USA) resulting in a final eluate volume of 20 μL. The cfTNA was loaded onto the Genexus™ integrated sequencer, and run using the Oncomine precision assay GX, an amplicon-based panel of 50 genes including DNA hotspots and RNA fusions. This assay includes automated library preparation, sequencing, and bioinformatic analysis (Genexus Software 6.2.2, Thermofisher, Waltham, MA, USA) [19].

### 2.3. Chart Review

To assess the early clinical experience, a retrospective chart review of liquid biopsies performed at William Osler Health System, Canada, between the period January 2021 and November 2023 was conducted. Study approval was provided by the William Osler Health System research ethics board (REB#0118).

Cases were identified using clinical sequencing logs and associated electronic medical records were reviewed. Information collected included demographic and pathology data, as well as the investigative and therapeutic course of the patient’s cancer care journey. An effort to ascertain the underlying reason for the liquid biopsy was made by the study team.

Liquid biopsy reports were reviewed and assessed for concordance where a matched tissue biopsy was available. The determination of whether a tissue biopsy was either matched or concordant was made by the study team upon retrospective review, if not explicitly stated in the liquid biopsy report at the time of interpretation.

Turnaround time was defined as the blood-draw date to the molecular report sign-out date, reported in “business days” by removing weekends and statutory Canadian holidays.

## 3. Results

From January 2021 to November 2023, a total of 124 consecutive patients underwent liquid biopsy testing. The median age was 67 years (range 23–94 years), with 68 (55%) females. This cohort had predominantly advanced-stage patients, including 3 (2%) with stage III, 114 (92%) with stage IV cancer, and 7 (6%) with unknown staging. The majority of patients (69% (*n* = 86)) had a lung cancer diagnosis, followed by gastrointestinal malignancies (13% (*n* = 16)) and breast cancer (10% (*n* = 13)). Among patients with lung cancer, 55 (64%) of them had no tobacco use history, and 77 (90%) had an adenocarcinoma histologic subtype. Additional demographic data are summarized in Table 1.

From the same time period, same oncology population, and same clinical practitioners, 1759 tissue-based NGS assays were performed in-house.

**Figure 1:**
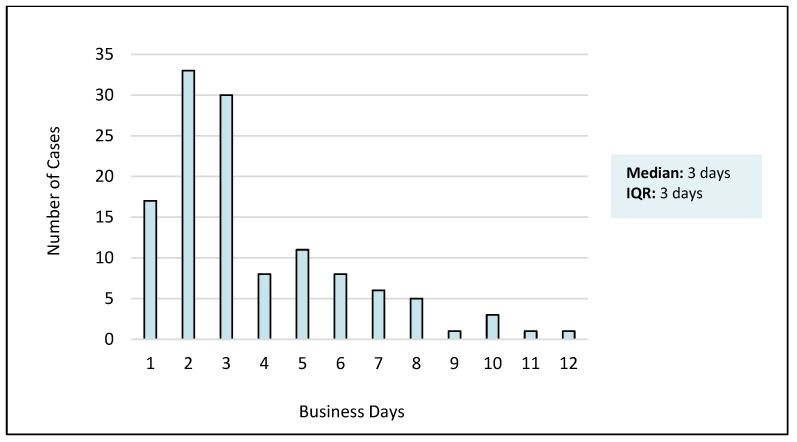
Turnaround time of liquid biopsy molecular results, reported in business days.

The median turnaround time for liquid biopsy was 3 business days (interquartile range 2–5 business days). A total of 17 (14%) cases had a turnaround time of 1 day, and 80 cases (65%) were reported in 3 days or less. Turnaround time data are summarized in Figure 1.

**Figure 2:**
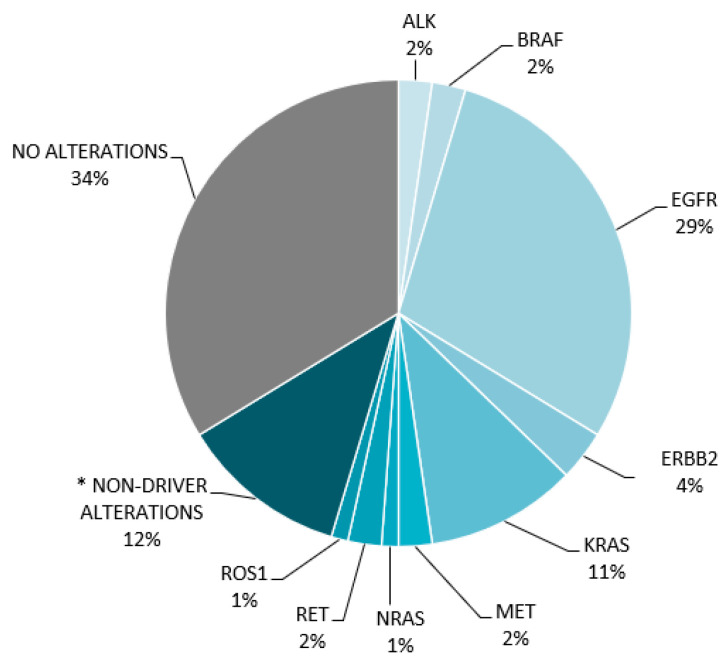
Distribution of driver mutations in lung cancers (*n* = 86). * Non-driver mutations include *TP53* and *PIK3CA*, among others.

**Figure 3:**
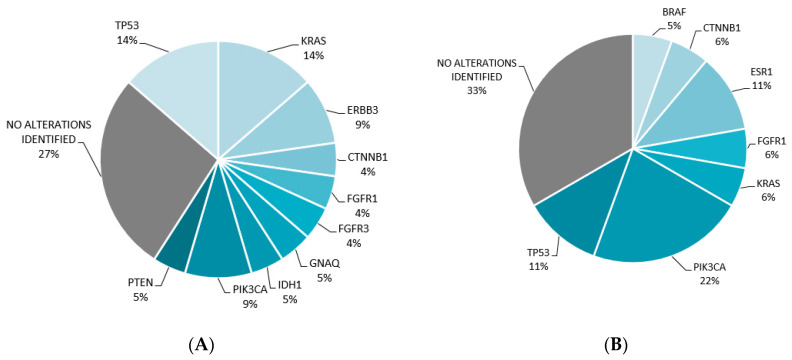
Distribution of mutations. (**A**) Total mutations (*n* = 22) identified in gastrointestinal cancers (*n* = 16). (**B**) Total mutations (*n* = 18) identified in breast cancers (*n* = 13).

Of the 124 sequenced plasma samples, 77 (62%) cases reported alterations and 38% of the cases had no alterations (either negative or nondiagnostic). Among the identified mutations were single nucleotide variants and insertions/deletions (86%), copy number alterations (9%), and fusions (5%). *EGFR* was the most commonly identified driver mutation in non-small cell lung cancer (29% of cases, *n* = 86). In gastrointestinal malignancies, *KRAS* mutation was found in 14% of cases (*n* = 16), and in breast malignancies, *PIK3CA* was identified in 22% of cases (*n* = 13), see Figure 2 and Figure 3. 

Overall, 75 cases (61%) had a matched tissue result available for comparison, and a driver alteration was present in 68 (91%) of these cases. Fifty-three (71%) matched tissue and liquid cases were concordant, with 22 cases (29%) demonstrating a driver mutation in a tissue sample only with a false-negative liquid biopsy result. There were no cases (0%) of liquid biopsy with matched tissue biopsy where a driver mutation was found in the liquid biopsy and not in the tissue sample.

Looking separately at alterations identified by ctDNA (SNVs, INDELs, and CNAs), 57 driver alterations were identified, 41 (72%) were detected by both tissue and liquid biopsy, and 16 (28%) were identified by tissue biopsy only. For gene fusions, detected by ctRNA sequencing, 11 cases were identified with matched tissue biopsy. Fusion drivers were detected in both tissue and plasma in five cases (46%), and in a tissue sample only in six cases (55%).

Seven cases showed no driver alteration on tissue testing, all of these cases were driver-negative on liquid biopsy. MET exon 14 skipping alterations could be detected either by ctDNA as an SNV/INDEL or by ctRNA as a MET(13)::MET(15) fusion. Three cases of MET exon 14 skipping were identified with matched tissue samples, the ctDNA alteration was identified in three of three cases (100%), whereas the accompanying ctRNA fusion was identified in one of three cases (33%).

Comparison of liquid biopsy to matched tissue NGS results, where available, are summarized in Table 2.

Actionable driver mutations were identified in eight liquid biopsies (6.5%) where no concurrent tissue sample was available for testing.

**Figure 4:**
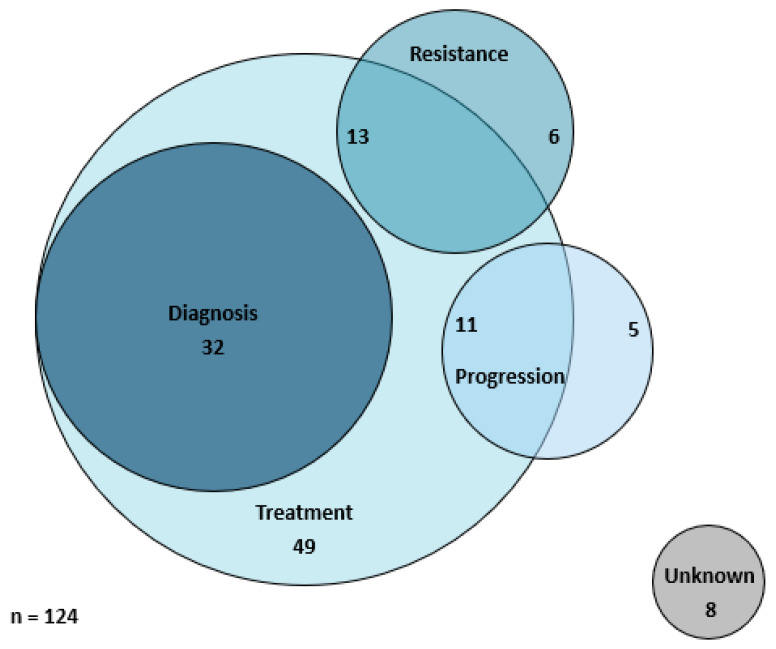
Utilization patterns of liquid biopsy. Of 124 liquid biopsies, 32 were initiated to inform diagnoses and treatment investigations, 13 to inform resistance and treatment investigations, 11 to inform progression and treatment investigations, 49 to inform treatment investigations only, 6 to inform resistance investigations only, 5 to inform progression investigations only, and 8 for unknown reasons.

As liquid biopsy was available in an unrestricted fashion, user’s intent was extracted from the medical record. The most common reason for performing a liquid biopsy was to guide treatment in 105 cases (85%). Additional uses included: establishing a diagnosis in 32 cases (19%), determining resistance mechanisms to a targeted therapy in 19 cases (11%), as well as determining if a patient’s cancer was progressing on their current non-targeted (chemo- or immunotherapy) therapy in 16 cases (9%). The intent of the liquid biopsy often overlapped, see Figure 4.

In 32 cases where the clinical user was attempting to make a primary cancer diagnosis, 21 (66%) of these liquid biopsies yielded mutational information, and in four cases (13%) a diagnosis was made by liquid biopsy without further invasive tissue sampling.

## 4. Discussion

Liquid biopsy has emerged as a powerful tool in precision cancer care, offering considerable advantages over, and nicely complementing, tissue-based biomarker testing. This study demonstrates the feasibility and clinical utility of a rapid in-house point of care style technique.

Over an approximately 3-year period, 124 liquid biopsies were reported with a median turnaround time of 3 days. This result is in keeping with previous reports using similar methods [20]. To our knowledge, at the time of this writing, this represents the fastest turnaround time reported for comprehensive liquid NGS. With this study, adding to previous reports, a rapid in-house approach offers a considerable time advantage over the use of commercial labs [21,22,23].

The timeliness of treatment is critical in diseases such as non-small cell lung cancer, where the mortality of untreated advanced disease is 4% per week, and the cost to the healthcare system in providing supportive care is over $400 dollars per week [24,25]. Treatment initiation without biomarker data can have a severely adverse effect on patient outcomes [26]. The median 3-day turnaround time reported here for liquid biopsy, and previously for tissue biopsy, represents practice-changing advances in the delivery of biomarkers to support precision cancer care as a standard practice in community settings [17]. Variability in the turnaround time (range 1–12 days) was due to technologist and sequencing instrument availability. In-house biomarker testing is prioritized according to patient needs, with faster turnaround times provided when indicated. In this series, 14% of liquid biopsies were reported within 24 h of blood draw to accommodate stat and urgent requests. 

Negative liquid biopsies found no results. Non-diagnostic liquid biopsies failed to detect a known driver mutation or failed to meet appropriate quality metrics. It is often difficult to distinguish a non-diagnostic sample from a negative sample, especially in scenarios with no prior biomarker information available. As liquid biopsy uptake continues, clinician users may benefit from support around the interpretation and appropriate response to results, particularly around negative/nondiagnostic tests.

The overall concordance of liquid biopsy to matched tissue results was relatively high; however, this was driven largely by point mutations and other alterations identified in ctDNA. The liquid biopsy assay utilizes ctRNA to identify fusions. This performed relatively poorly with an overall sensitivity of only 46% compared to matched tissue results. The results suggest that this is not a reliable method for the detection of oncogenic fusions in plasma. The phenomenon is nicely demonstrated by MET exon 14 skipping alterations which can be detected in both the ctDNA and ctRNA components of the panel. Here we reported three patients, identified by matched tissue biopsy, to harbor a MET exon 14 skipping driver mutation. All three were detected in the form of a ctDNA splice site alteration; however, only one of the three was concurrently detected in the ctRNA space as a MET(13)::MET(15) fusion. Typically, tissue-based NGS shows the opposite pattern, with RNA sequencing demonstrating a higher sensitivity [27].

The majority of commercially available liquid biopsy assays utilize a ctDNA-only approach. In the plasma space, reports from ctDNA hybrid capture-based approaches have shown superior sensitivity for the detection of ALK fusions [28]. Previous studies have shown the Oncomine precision assay to have a high sensitivity for fusions, particularly at low RNA concentrations [29]. In the setting of liquid biopsy, the paradoxically low sensitivity for fusions is believed to be pre-analytic in nature.

Future improvements to blood collection and transport, as well as ctRNA extraction from plasma, may improve upon the sensitivity. While Streck blood collection tubes and transport of peripheral blood in ambient temperature do not impact ctDNA, it is likely to affect ctRNA viability [30]. As such, blood collection tubes that can also preserve ctRNA and temperature-controlled transport of peripheral blood may be beneficial; however, further research is required. Until that time, users should be aware of this limitation, particularly in diseases where oncogenic fusions are relevant to diagnosis and treatment. The low sensitivity for fusions seen in this approach may be balanced against the ease of use, implementation, and rapid turnaround time associated with this methodology. 

This study was conducted in a public healthcare setting, where cost remains front of mind, and the price for liquid biopsy is a prominent disincentive for payers. It should be viewed as a very positive signal that over an approximate 3-year study period, 124 liquid biopsy tests were ordered—none were rejected, and no limitations were placed on usage. For comparison, over this same study period, 1759 tissue-based NGS were performed for the same oncology practice group, with liquid biopsy representing less than 7% of all NGS usage. Within many healthcare systems, faster results are often quoted as an underpinning reason for the use of liquid biopsy [2,3]. Within our center, during this study period, rapid tissue-based NGS was also available for patients and providers, and hence within the usage data, liquid biopsy for “faster results” was not observed. In our center, a setting with rapidly-available tissue results, liquid biopsy was requested fewer than 50 times per year or less than once per week. This would represent a limitation of this study as results may not be transferrable to community centers with protracted wait times for biomarkers, or even those with standard 14-day wait times.

Within the usage data, there were two usages identified, which have not previously been well described for liquid biopsy in cancer care. In this series, users attempted to make a primary cancer diagnosis in 32 liquid biopsies. All of these instances would represent a patient with suspected cancer, in whom a tissue diagnosis was either not possible, challenging, or too time-consuming. Additionally, many liquid biopsies were ordered to diagnose progression (outside of acquired resistance to a targeted therapy). In these cases, clinicians would use liquid biopsy data to distinguish cancer progression from mimics such as radiation pneumonitis, infection, and others. These utilizations of liquid biopsy are certainly facilitated by the rapid turnaround time associated with the technique and may not be possible with a turnaround time of 2 weeks or greater. As discussed above, the integration of other available data, including imaging, patient history, and multidisciplinary tumor board discussion is invaluable to the interpretation of liquid biopsy when looking beyond the elucidation of matched targeted therapies.

In this cohort, 21 patients were diagnosed with cancer, and some may have avoided an invasive sampling procedure, associated complications, and tissue-based testing costs and delays. In four cases, a diagnosis was made without a tissue biopsy. The systemic cost savings of in-house liquid biopsy implementation is felt to be large, but further studies are needed to quantify this. The utility in this regard will also likely improve with both clinician and laboratorian experience. While some patients were able to avoid invasive procedures, the utility of tissue biopsy including morphology, histology, and immunohistochemistry remains paramount in precision cancer care.

Many studies have demonstrated the complementary nature of tissue and liquid biopsies, showing that while many actionable genomic alterations are shared between tissue and liquid testing, many are private to either tissue-only or liquid-only [31,32,33,34]. In this cohort, there were no instances where liquid biopsy identified a mutation that was not detected by matched tissue sequencing; however, alterations were identified by plasma-only where a tissue biopsy was unavailable. This dataset is intended to highlight the value of concurrently available rapid point of care liquid and tissue-based NGS in a single center.

## 5. Conclusions

Rapid in-house liquid biopsy is feasible and can be implemented in community practice to support cancer care. This point of care technique enables a median turnaround time of 3 business days. The technique offers considerable advantages over tissue biopsy alone, but may be associated with lower sensitivity, especially for gene fusions. This study highlights additional clinical utility beyond targeted therapy selection, such as primary cancer diagnosis.

Liquid biopsy is proven to be a valuable test in cancer care. A rapid in-house approach offers considerable advantages over more traditional send-out methods. More work is needed to improve the sensitivity of ctRNA-based NGS to detect oncogenic fusions. Additional studies are needed to measure the economic value and cost effectiveness of the technique.

## Figures and Tables

**Table 1:** Patient demographic and baseline clinical characteristics.

Characteristics				*n* = 124 (%)
**Age, years**				
	Median (range)			67 (23–94)
**Sex**				
	Female			68 (55)
	Male			56 (45)
**ECOG**				
	0			18 (15)
	1			66 (53)
	2			24 (19)
	3			8 (6)
	4			2 (2)
	Unknown			6 (5)
**Stage**				
	III			3 (2)
	IV			114 (92)
	Unknown			7 (6)
**Tumor site**				
	Breast			13 (10)
	Gastrointestinal			16 (13)
	Other			9 (7)
	Lung			86 (69)
		**Smoking status (lung cohort)**		*n* = 86 (%)
			Non-smoker	55 (64)
			Current/former smoker	26 (30)
			Unknown	5 (6)
		**Histologic subtype (lung cohort)**		*n* = 86 (%)
			Adenocarcinoma	77 (90)
			SCC	3 (3)
			NOS	6 (7)

SCC—squamous cell carcinoma; NOS—not otherwise specified.

**Table 2:** Comparison of driver alterations between liquid biopsies to matched tissue results.

Genomic Alteration		Total Cases with Matched Tissue	Mutations Found in Tissue and Plasma	Mutations Found in Tissue Only
**SNVs, INDELs, CNVs (DNA)**		**n**	**n (%)**	**n (%)**
	BRAF	1	1	-
	EGFR	31	23	8
	ERBB2	3	3	-
	ERBB4	1	-	1
	KRAS AMPLIFICATION	1	1	-
	KRAS POINT MUTATION	13	7	6
	MET AMPLIFICATION	1	-	1
	MET SPLICING MUTATION	3	3	-
	PIK3CA	3	3	-
*Total DNA alterations*		57	41 (72%)	16 (28%)
**FUSIONS (RNA)**		**n**	**n (%)**	**n (%)**
	ALK	4	2	2
	FGFR1	1	1	-
	MET FUSION	3	1	2
	NTRK	1	-	1
	RET	1	-	1
	ROS1	1	1	-
*Total RNA alterations*		11	5 (46%)	6 (55%)
	NO ALTERATIONS IDENTIFIED	7	7 (100%)	
*Total alterations*		75	53 (71%)	22 (29%)

SNV—single nucleotide variants; INDELs—insertion/deletion mutations; CNV—copy number variants.

## Data Availability

Supporting data can be available from the corresponding author, B.S.S., upon reasonable request.
